# Prediction of distant metastases at diagnosis in medullary thyroid cancer: combining calcitonin with lymph node imaging

**DOI:** 10.1210/jendso/bvag002

**Published:** 2026-01-08

**Authors:** Leonoor E Schonebaum, Hossein Razaghi Siahroudi, Layal Chaker, Sjoerd A A van den Berg, Tessa M van Ginhoven, Robin P Peeters, W Edward Visser

**Affiliations:** Academic Center for Thyroid Disease, Department of Internal Medicine, Erasmus Medical Center, Rotterdam 3015 CE, The Netherlands; Academic Center for Thyroid Disease, Department of Internal Medicine, Erasmus Medical Center, Rotterdam 3015 CE, The Netherlands; Academic Center for Thyroid Disease, Department of Internal Medicine, Erasmus Medical Center, Rotterdam 3015 CE, The Netherlands; Department of Epidemiology, Erasmus University Medical Center, Rotterdam 3015 CE, The Netherlands; Diagnostic Laboratory for Endocrinology, Department of Internal Medicine, Erasmus Medical Center, Rotterdam 3015 CE, The Netherlands; Department of Clinical Chemistry, Erasmus Medical Center, Rotterdam 3015 CE, The Netherlands; Academic Center for Thyroid Disease, Department of Oncological Surgery, Erasmus Medical Center Cancer Center, Rotterdam 3015 CE, The Netherlands; Academic Center for Thyroid Disease, Department of Internal Medicine, Erasmus Medical Center, Rotterdam 3015 CE, The Netherlands; Academic Center for Thyroid Disease, Department of Internal Medicine, Erasmus Medical Center, Rotterdam 3015 CE, The Netherlands

**Keywords:** medullary thyroid cancer, diagnosis, calcitonin, distant metastases, imaging, lymph nodes

## Abstract

**Context:**

Current guidelines recommend performing preoperative additional imaging in patients with medullary thyroid cancer (MTC) when calcitonin levels exceed 500 pg/mL to detect distant metastases (M1). However, this recommendation is based on limited evidence. Whether this is the optimal cutoff, including other diagnostic characteristics, has only been partially evaluated in research.

**Objective:**

The aims of this study were to evaluate the current calcitonin-driven recommendation and to investigate whether other diagnostic characteristics can be added to improve prediction of M1 disease at diagnosis.

**Methods:**

Adult MTC patients treated in a tertiary care hospital between 1984 and 2023 with a preoperative calcitonin measurement were retrospectively collected. M1 disease was detected by preoperative imaging or biopsy. Logistic regression was used to identify new predictors for M1 at diagnosis.

**Results:**

In total, 81 patients with MTC were included for analysis. M1 disease at presentation was found in 27%. Sensitivity and specificity for the current calcitonin cutoff were 90.9% (95% CI, 72.2-97.5) and 47.5% (95% CI, 35.3-60.0), respectively. In multivariable analysis, presence of suspicious lymph nodes on preoperative ultrasound was the strongest predictor (odds ratio [OR] 6.7; 95% CI, 1.3-34.2; *P* = .022) followed by calcitonin (OR 1.9; 95% CI, 1.2-2.8; *P* = .005) for M1 disease.

**Conclusion:**

To our knowledge, this is the first study investigating the optimal combination of predictors for M1 disease in MTC at diagnosis. Suspicious lymph nodes on ultrasound is the strongest predictor for M1 disease, well exceeding calcitonin levels. Incorporating suspicious lymph nodes and calcitonin into a novel clinical decision tool may optimize M1 detection while reducing unnecessary imaging.

Medullary thyroid carcinoma (MTC) is a neuroendocrine tumor derived from the calcitonin-producing parafollicular C-cells of the thyroid gland. MTC comprises 1% to 3% of all thyroid carcinomas. Compared to differentiated thyroid carcinoma, MTC more often presents with metastasized disease and has a higher mortality rate in case of extrathyroidal disease [1-3]. However, when only localized disease is present, curative treatment is possible and long-term survival can be achieved. Approximately 25% of MTCs occur in the form of a hereditary syndrome, namely multiple endocrine neoplasia type 2A (MEN2A), MEN2B or familial MTC (FMTC) [4]. In the remaining 75%, the disease is sporadic. A substantial number of patients present with already metastasized disease: 35% with cervical lymph node metastasis and 5% to 15% with distant metastasis [5-8].

The cornerstone of treatment for MTC is a total thyroidectomy with central compartment lymph node dissection. The specific treatment approach, including the extent of the initial surgery and the intention of treatment being curative or palliative, depends on the stage of the disease at the time of diagnosis and mostly on the question whether the disease has spread beyond the thyroid. The presence of distant metastases (M1) at diagnosis is generally assessed using multiple imaging modalities, including contrast-enhanced computed tomography (CT) scan of neck and chest, liver and axial magnetic resonance imaging (MRI), bone scintigraphy and [^18^F]-fluorodeoxyglucose (FDG) or [^18^F]- fluoro-dihydroxyphenylalanine (FDOPA)–positron emission tomography (PET) [9, 10].

Unnecessary imaging in patients without distant metastases can potentially be harmful. First, it imposes a burden on patients due to the necessity of extra hospital visits and the anxiety of waiting for the results. Second, imaging can result in incidental findings leading to other diagnostic procedures and treatments, which in some cases may pose an additional risk to the patient and have the potential to delay the decision of the final treatment plan. Third, the expenses associated with these imaging tests are considerable, underscoring the importance of keeping cost-effectiveness in mind. Nevertheless, it is crucial not to overlook distant metastases in patients in whom their presence would necessitate a different treatment.

Currently, the decision to perform additional imaging to detect possible distant metastases is mainly guided by preoperative serum calcitonin levels. The 2015 American Thyroid Association (ATA) guideline recommends performing additional imaging when serum calcitonin levels exceed 500 pg/mL [9]. However, this recommendation is based on a single study that investigated the correlation between calcitonin and the extent of lymph node metastases [11]. Whether this is the optimal cutoff has only partially been evaluated in one other study [12]. Moreover, the predictive value of other diagnostic characteristics, such as lymph node status and tumor size on preoperative imaging, has not been previously investigated.

Therefore, the aims of this study were 2-fold: (1) to assess the suggested calcitonin cutoff of 500 pg/mL; and (2) to evaluate the current ATA guideline recommendation and to investigate whether additional diagnostic characteristics can be added to improve prediction of M1 disease at diagnosis.

## Methods

### Study population and data collection

We retrospectively collected a cohort of adult patients diagnosed and/or treated for MTC in our academic hospital (Erasmus Medical Center, Rotterdam, the Netherlands) between 1984 and 2023. We included all patients with a preoperative serum calcitonin measurement and at least 1 year of follow-up after the date of diagnosis. Patients with initial presentation attributable to distant metastases were excluded, as they follow a different diagnostic trajectory. Information on demographic characteristics and disease-related data were obtained from electronic patient records. We collected the following variables: age at diagnosis, sex, hereditary status, preoperative calcitonin and carcinoembryonic antigen (CEA) levels, the presence of suspicious lymph nodes on preoperative ultrasound (US), the presence of pathologically proven lymph node metastases, tumor size measured on preoperative imaging, the presence of distant metastases and response to treatment. Hereditary status was determined by the presence of germline rearranged during transfection (RET) mutations. For 3 patients without information on germline RET mutations, sporadic status was assumed based on a negative family history for MTC and MEN2-related tumors together with the absence of other clinical signs of MEN2 syndrome. The presence of suspicious lymph nodes and tumor size were evaluated with preoperative US. If US did not report tumor size, preoperative contrast-enhanced CT scan was used to assess this variable. Suspicious lymph nodes on US refer to central as well as cervical nodes. In accordance with the Dutch guidelines, lymph nodes were considered to be suspicious for malignancy if at least one of the following characteristics was present: microcalcifications, partially cystic appearance, peripheral or diffusely increased vascularization, hyperechoic tissue, round shape, or absence of the hilum. Fine needle aspiration (FNA) is performed on all lymph nodes deemed suspicious. Lymph nodes were classified as pathologically confirmed metastases when FNA or biopsy revealed malignant cells.

Response to treatment was defined as previously described by Tuttle and Ganly [13]. No evidence of disease (NED) was characterized by undetectable calcitonin and normal range CEA in the absence of structurally identifiable disease. A biochemical incomplete response was defined as a detectable calcitonin or elevated CEA without structurally identifiable disease. Structural incomplete response referred to the presence of recurrent or persistent structurally identifiable disease either local or distant.

The study was approved by the Medical Ethics Committee of the Erasmus Medical Center (MEC 2012-561).

### Primary outcome

The primary outcome for this study was M1 disease at diagnosis. The presence of M1 disease was assessed during the diagnostic trajectory by at least one of the following imaging modalities: contrast-enhanced CT scan, MRI, and FDG-PET scan. M1 disease detected within the first 6 months of follow-up was assumed to be present at diagnosis and was therefore classified as M1. All other patients were classified as M0. In case preoperative imaging was not performed due to a preoperative calcitonin level below 500 pg/mL, the absence of M1 disease was checked by assessing the response to treatment and first year follow-up. The presence of M1 disease is very unlikely when there is NED within the first year of follow-up. Patients with a biochemical incomplete response underwent postoperative imaging to check for M1 disease.

### Measurement of biomarkers

Preoperative serum calcitonin levels were measured using Immulite 2000XPi (Siemens, The Hague, Netherlands), except for 7 samples collected before 2006 that were analyzed using radioimmunoassay (RIA). The lower limit of detection was 2 pg/mL and the normal range <10.5 pg/mL and <7.3 pg/mL for men and women, respectively. Preoperative serum CEA levels were measured using electrochemiluminescence immunoassay on a Cobas E801 analyzer (Roche Diagnostics, Penzberg, Germany, upper normal limit 5.0 ng/mL).

### Statistical analysis

For continuous variables, means and SD or medians with interquartile range (IQR) were calculated. Categorical variables were reported as absolute numbers with percentages. To analyze differences between groups, unpaired *t* test, Chi-square test, or Kruskal-Wallis test were used, depending on the type of variable. Performance of calcitonin was visualized by receiver operating characteristic (ROC) curve and analysis of area under the curve (AUC). Sensitivity, specificity, positive predictive value (PPV) and negative predictive value (NPV) with the corresponding 95% CI were calculated for different cutoffs. The association of potential predictors with M1 disease at diagnosis was studied using univariable and multivariable logistic regression. The linearity assumption was checked using restricted cubic splines (with 3 knots). All predictors were assessed for outliers and multicollinearity. Due to the large scale and presence of outliers, calcitonin and CEA levels were log-transformed, and calcitonin values were winsorized. Potential interaction between preoperative calcitonin and the presence of suspicious lymph nodes was examined. We used multiple imputation for predictors with missing data (hereditary status 1%; tumor size 7%). Five imputed datasets were created and pooled for analyses using R package “mice.” To develop an optimal prediction model for M1 disease at diagnosis, backward selection was performed. Beginning with a model that included the most promising predictors from univariable analysis based on odds ratio (OR) and *P* value, the least significant predictor was eliminated step by step until the final model was obtained. During each step, model performance was assessed by checking the AUC value and R2 (Nagelkerke). The confidence interval of the final model was calculated using the pROC package with 2000 bootstrap simulations. A sensitivity analysis was conducted excluding all samples collected before 2006. Statistical analysis was performed using R statistical software (version 4.3.2) and GraphPad Prism version 9.0.0 (GraphPad Software, San Diego, CA, USA). A *P* value <.05 was considered statistically significant.

## Results

### Characteristics of the study population

From a total cohort of 130 patients with MTC, 81 patients could be included for analyses (Fig. S1) [14]. Reasons for exclusion were no preoperative calcitonin measurement (n = 32); follow-up of 1 year not yet reached (n = 2); data on first year of follow-up missing (n = 1); age <18 years (n = 2); and first presentation attributable to distant metastases (n = 12). Mean age was 55.1 (± 15.5) years, 49% were female (n = 40) and 76% had sporadic MTC (n = 61). M1 disease at diagnosis was found in 27% (n = 22) of all patients. These patients had significantly higher preoperative calcitonin measurements than the M0 group (4441.5 pg/mL, IQR 1367.0-14814.5 vs 647.0 pg/mL, IQR 122.5-1743.5, *P* < .001). Furthermore, the M1 group had larger tumor size (3.4 cm, IQR 2.1-4.6 vs 1.6 cm, IQR 1.1-3.1, *P* = .002) and more often suspicious lymph nodes on US (90.9% vs 42.4%, *P* < .001). A total of 20 patients did not undergo preoperative screening for M1 disease because their calcitonin levels were below 500 pg/mL. Of these, 17 remained disease-free during follow-up, while 3 had a biochemical incomplete response with no evidence of M1 disease on additional imaging. Suspicious central lymph nodes were present in only 6 cases. In each of these 6 cases, there were also suspicious lateral lymph nodes present upon the US. See Table 1 for all baseline characteristics.

**Table 1:** Demographic characteristics of the study population

	Total population	M0	M1	*P* value*^a^*
Characteristic	(n = 81)	(n = 59)	(n = 22)	
Age at diagnosis (years)	55.1 (15.5)	55.2 (15.0)	54.9 (17.0)	.931
Sex				.495
Female	40 (49.4)	31 (52.5)	9 (40.9)	
Male	41 (50.6)	28 (47.5)	13 (59.1)	
Hereditary status				.109
Sporadic disease	61 (76.2)	41 (70.7)	20 (90.9)	
MEN2	19 (23.8)	17 (29.3)	2 (9.1)	
Preoperative calcitonin, pg/mL	1272.0 (178.0-3000.0)	647.0 (122.5-1743.5)	4441.5 (1367.0-14814.5)	<.001
Preoperative CEA, ng/mL	50.0 (7.32-164.0)	15.2 (5.6-105.0)	180.0 (42.6-264.8)	<.001
Suspicious N on US				<.001
Absent	36 (44.4)	34 (57.6)	2 (9.1)	
Present	45 (55.6)	25 (42.4)	20 (90.9)	
Pathologically proven N				<.001
Absent	53 (65.4)	46 (78.0)	7 (31.8)	
Present	28 (34.6)	13 (22.0)	15 (68.2)	
Tumor size, cm	2.0 (1.2-3.7)	1.6 (1.1-3.1)	3.4 (2.1-4.6)	.002
Response to treatment				<.001
NED	26 (32.1)	26 (44.1)	0 (0.0)	
BiR	30 (37.0)	30 (50.8)	0 (0.0)	
SiR, local	3 (3.7)	3 (5.1)	0 (0.0)	
SiR, distant	22 (27.2)	0 (0.0)	22 (100.0)	

Values are means (± SD), medians (interquartile range), or numbers (percentages)

Abbreviations: BiR, biochemical incomplete response; CEA, carcinoembryonic antigen; M, distant metastases; MEN, multiple endocrine neoplasia; N, lymph node; N+, lymph node metastasis; NED, no evidence of disease; SiR, structural incomplete response; US, ultrasound.

^*a*^ *P*-values comparing M0 with M1

### Current guideline evaluation

**Figure 1:**
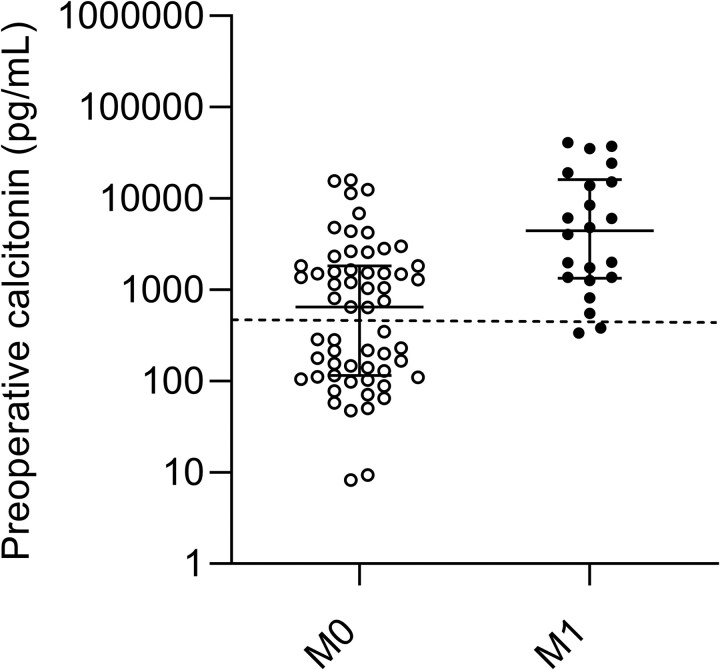
Scatter plot of preoperative calcitonin levels on a logarithmic scale in the M0 (open dots) vs M1 (closed dots) group. Error bars represent median with interquartile ranges. The dashed line represents the current calcitonin cutoff of 500 pg/mL.

First, we evaluated the current recommendation to perform additional imaging when calcitonin levels exceed 500 pg/mL. Figure 1 shows the preoperative calcitonin values in the study cohort. A large proportion of the samples in the M0 group (53%, 31/59) had calcitonin levels above 500 pg/mL. Following the current guideline, these patients underwent unnecessary imaging procedures. Also, 2 patients in the M1 group had a calcitonin level below the cutoff of 500 pg/mL, where M1 disease would have been missed if imaging was not performed. Sensitivity and specificity for the current calcitonin cutoff of 500 pg/mL were 90.9% (95% CI, 72.2-97.5) and 47.5% (95% CI, 35.3-60.0), respectively. Table 2 presents the diagnostic performance parameters of various calcitonin cutoffs. The AUC for calcitonin was 0.80 (95% CI, 0.69-0.89) (see Fig. S2 for the ROC curve) [14].

**Table 2:** Diagnostic performance of calcitonin for M1 at different cutoffs

Calcitonin cutoff	Sensitivity %	Specificity %	PPV %	NPV %	Positive LR	Negative LR
pg/mL	(95% CI)	(95% CI)	(95% CI)	(95% CI)	(95% CI)	(95% CI)
300	100.0 (85.1-100.0)	45.8 (33.7-58.3)	40.7 (28.7-54.0)	100 (87.5-100.0)	1.8 (1.6-2.2)	0.0 (0.0-0.0)
400	90.9 (72.2-97.5)	47.5 (35.3-60.0)	39.2 (27.0-52.9)	93.3 (78.7-98.2)	1.7 (1.4-2.2)	0.2 (0.1-0.3)
500	90.9 (72.2-97.5)	47.5 (35.3-60.0)	39.2 (27.0-52.9)	93.3 (78.7-98.2)	1.7 (1.4-2.2)	0.2 (0.1-0.3)
1000	81.8 (61.5-92.7)	54.2 (41.7-66.3)	40 (27.0-54.5)	88.9 (74.7-95.6)	1.8 (1.4-2.3)	0.3 (0.2-0.5)
2000	59.1 (38.7-76.7)	78.0 (65.9-86.6)	50 (32.1-67.9)	83.6 (71.7-91.1)	2.7 (1.9-3.8)	0.5 (0.4-0.7)

Abbreviations: LR, likelihood ratio; M1, distant metastases; NPV, negative predictive value; PPV, positive predictive value.

### Predictors for M1 disease

Second, we set out to identify new predictors for M1 disease at diagnosis. In the univariable analysis, preoperative calcitonin (OR 2.09; 95% CI, 1.41-3.09; *P* < .001), preoperative CEA (OR 1.67; 95% CI, 1.20-2.33; *P* = .003), both the presence of suspicious lymph nodes on US and pathologically proven lymph node metastases (OR 13.60; 95% CI, 2.84-65.18; *P* < .001 and OR 7.58; 95% CI, 2.51-22.90; *P* < .001, respectively) and a larger tumor size (OR 1.68; 95% CI, 1.19-2.36; *P* = .003) were all significantly associated with M1 disease at diagnosis. Categorizing tumor size (>3 cm vs ≤3 cm) resulted in an even larger OR of 4.74 (95% CI, 1.60-14.00; *P* = .006). See Table 3 for all predictors. There was no significant interaction between preoperative calcitonin and the presence of suspicious lymph nodes.

**Table 3:** Univariable and multivariable logistic regression of different predictors for M1 at diagnosis

	OR		OR	
Predictor	(95% CI)	*P* value	(95% CI)	*P* value
Age at diagnosis, years	1.00 (0.97-1.03)	.930		
Sex, male	1.60 (0.58-4.38)	.356		
Sporadic disease	4.05 (0.83-19.72)	.083		
Preoperative calcitonin, pg/mL*^a^*	2.09 (1.41-3.09)	<.001	1.85 (1.20-2.84)	.005
Preoperative CEA, ng/mL*^a^*	1.67 (1.20-2.33)	.003		
Suspicious N on US	13.60 (2.84-65.18)	.001	6.71 (1.32-34.18)	.022
Pathologically proven N+	7.58 (2.51-22.90)	<.001		
Tumor size, cm	1.68 (1.19-2.36)	.003		
Tumor size >3 cm*^b^*	4.74 (1.60-14.00)	.006		

Backward selection resulted in a multivariable model consisting of preoperative calcitonin levels and the presence of suspicious lymph nodes on imaging with an AUC of 0.85 (95% CI of 0.75-0.93).

Abbreviations: AUC, area under the curve; M, distant metastasis; N, lymph node; N+, lymph node metastasis; OR, odds ratio; US, ultrasound.

^*a*^log-transformed.

^*b*^Categorized tumor size (>3 cm vs ≤3 cm).

Backward selection using the most promising predictors from univariable analysis resulted in a multivariable model consisting of 2 predictors, namely the preoperative calcitonin level and the presence of suspicious lymph nodes on US. The multivariable model had a good discriminative ability for the identification of M1 disease at diagnosis with an AUC of 0.85 (95% CI, 0.75-0.93). The presence of suspicious lymph nodes remained the strongest predictor in the multivariable model (OR 6.71; 95% CI, 1.32-34.18; *P* = .022), followed by preoperative calcitonin (OR 1.85; 95% CI, 1.20-2.84; *P* = .005). Inclusion of categorized tumor size in the multivariable model resulted in a slightly higher AUC of 0.88 (95% CI, 0.79-0.95; see Table S1) [14]. However, due to the limited sample size and number of events, this study is likely statistically underpowered to robustly support a model with 3 variables.

**Figure 2:**
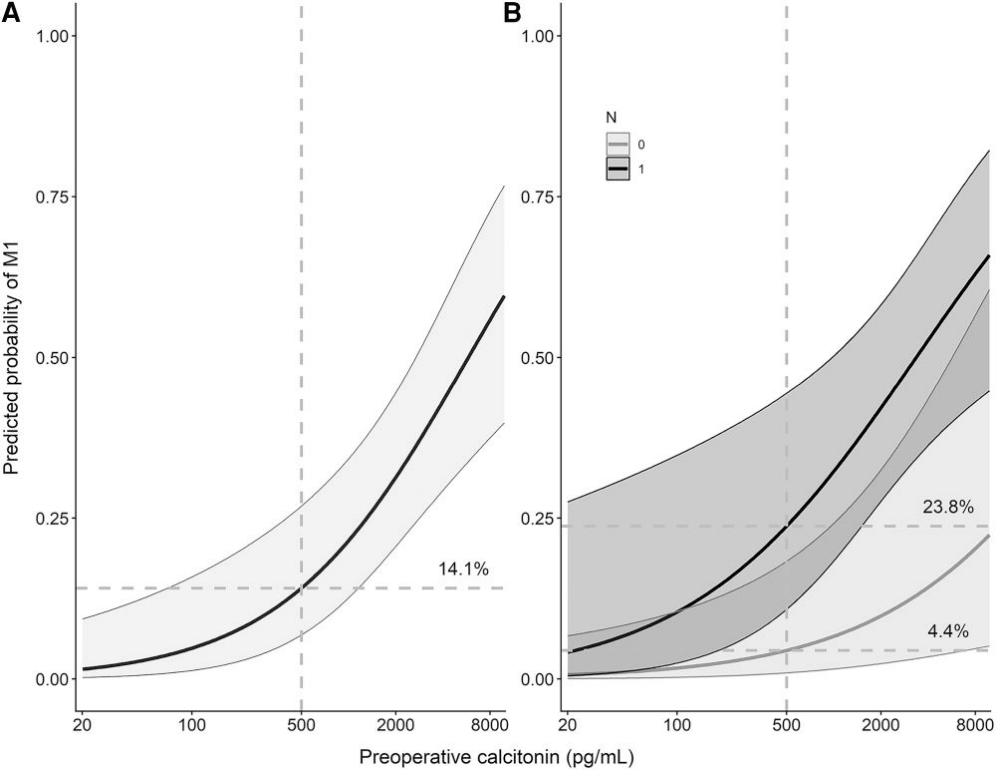
Predicted probability of M1 at diagnosis based on a univariable model with calcitonin levels alone (A) or a multivariable model including calcitonin levels and the presence of suspicious lymph nodes on ultrasound (B). The vertical gray dashed lines represent the current calcitonin cutoff of 500 pg/mL. The horizontal gray dashed lines represent the predicted probability of M1 at that cutoff: 14.1% (95% CI, 6.8-26.9) for the univariable model. In the multivariable model, the probabilities were 23.8% (95% CI, 10.8-44.4) and 4.4% (95% CI, 1.0-18.3) for patients with or without suspicious lymph nodes (N1 vs N0), respectively. *The x-axis is on a logarithmic scale, with tick marks representing normal calcitonin values.

We conducted a sensitivity analysis excluding all samples collected before 2006, which did not yield different results (multivariable model with AUC of 0.86 [95% CI, 0.77-0.94]; preoperative calcitonin OR 1.92 [95% CI, 1.22-3.01; *P* = .005]; suspicious lymph nodes, OR 7.39 [95% CI 1.41-38.70, *P* = .018]). Figure 2 shows the predicted probabilities of M1 disease at diagnosis for a univariable model with preoperative calcitonin levels alone and for the multivariable model. The predicted probability of M1 disease at diagnosis with a calcitonin level of 500 pg/mL was 14.1% (95% CI, 6.8-26.9) for the univariable model. In the multivariable model, the probability was 4.4% (95% CI, 1.0-18.3) for patients without suspicious lymph nodes (N0) and 23.8% (95% CI, 10.8-44.4) for those with suspicious lymph nodes (N1). The multivariable model demonstrates a clear distinction for the probability of M1 disease between patients with and without suspicious lymph nodes. An imaginary patient in our cohort with a calcitonin level of 400 pg/mL and suspicious lymph nodes has a 21.4% (95% CI, 9.0-42.8) predicted probability of M1 disease at diagnosis, whereas a patient with a calcitonin of 2000 pg/mL and no suspicious lymph nodes has a predicted probability of M1 of only 9.8% (95% CI, 2.4-32.5).

### Exploration of a potential new algorithm

**Figure 3:**
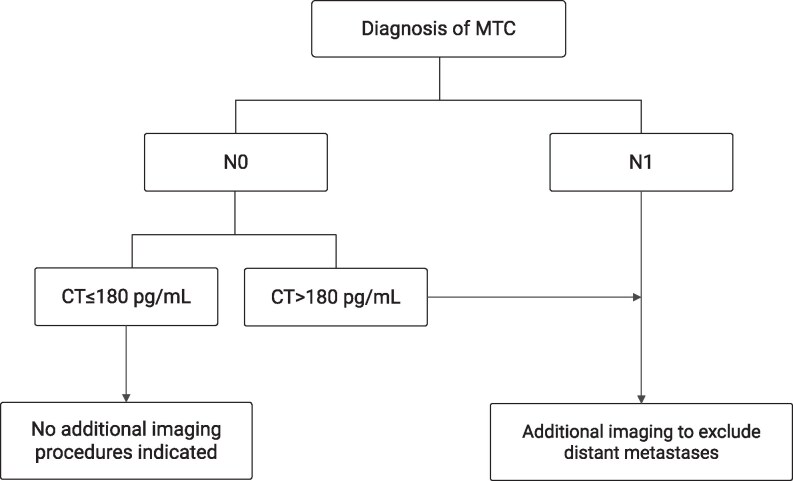
Potential novel decision tree corresponding to a predicted probability of M1 of 2.5% combining suspicious lymph nodes on ultrasound and preoperative calcitonin levels as predictors.

In our study population, the current calcitonin threshold of 500 pg/mL corresponded to a predicted probability of M1 disease of already 14.1%. To reduce the risk of overlooking M1 disease, we evaluated the diagnostic performance of both the univariable and multivariable model using 2 lower probability cutoffs (see Table 4). At a 2.5% cutoff, both models achieved perfect sensitivity of 100% (95% CI, 85-100). However, the multivariable model demonstrated superior specificity (29%; 95% CI, 18-42) compared to the univariable model (3%; 95% CI, 0-12). Applying this revised threshold in clinical practice would imply performing additional imaging in all patients with suspicious lymph nodes on US as well as in those with calcitonin levels exceeding 180 pg/mL but without suspicious nodal findings (see Fig. 3).

**Table 4:** Diagnostic performance of the univariable and multivariable model for 2 predicted probability of M1 cutoffs

Predicted probability M1	Sensitivity %	Specificity %	PPV %	NPV %	Positive LR	Negative LR
Cutoff	(95% CI)	(95% CI)	(95% CI)	(95% CI)	(95% CI)	(95% CI)
Univariable model						
2.5%	100 (85-100)	3 (0-12)	28 (18-39)	100 (16-100)	1.0 (1.0-1.1)	0 (0-NaN)
5.0%	100 (85-100)	20 (11-33)	32 (21-44)	100 (74-100)	1.3 (1.1-1.4)	0 (0-NaN)
Multivariable model						
2.5%	100 (85-100)	29 (18-42)	34 (23-47)	100 (80-100)	1.4 (1.2-1.7)	0 (0-NaN)
5.0%	95 (77-100)	39 (27-53)	37 (24-51)	96 (79-100)	1.6 (1.3-2.0)	0.1 (0.0-0.8)

Univariable model: M1 ∼ preoperative calcitonin

Multivariable model: M1 ∼ preoperative calcitonin + suspicious lymph nodes on US

Abbreviations: LR, likelihood ratio; M1, distant metastases; NPV, negative predictive value; PPV, positive predictive value; US, ultrasound.

## Discussion

To the best of our knowledge this is the first study investigating the association of multiple preoperative predictors with M1 disease at diagnosis with the aim of improving the recommendation for performing additional imaging. The data from our center demonstrated that the presence of suspicious lymph nodes on preoperative US is the strongest predictor for having M1 disease at diagnosis, and not preoperative calcitonin level. Furthermore, for the first time, we provide a comprehensive evaluation of the diagnostic performance of the current recommendation for performing additional imaging using a calcitonin cutoff of 500 pg/mL. This cutoff resulted in a moderate sensitivity of 91% and a poor specificity of only 48%. Moreover, the PPV and positive likelihood ratio (LR) were low, 39% and 1.7, respectively. Since our study cohort had a higher prevalence of M1 disease at diagnosis (27%) compared to the 5% to 15% reported in literature [5-8], the PPV in other populations with a lower prevalence will likely be even poorer due to a larger proportion of false positives, leading to more patients undergoing unnecessary imaging. Conversely, the NPV in our cohort (93%) will be higher in lower-prevalence populations, resulting in fewer missed cases of M1 disease.

A possible explanation for the higher prevalence of M1 disease at diagnosis in our study cohort compared to previous reports is that, in the Netherlands, thyroid nodule incidentalomas are not routinely analyzed. In contrast, countries that analyze incidentalomas or countries with thyroid nodule screening programs will detect a higher number of small, non-metastasized MTCs. This hypothesis is supported by the fact that MTC incidence rates are generally higher in other countries compared to the Netherlands [15-17]. To date, there remains significant clinical variability in practices regarding the use of calcitonin measurement in patients with thyroid nodules as a tool for the early detection of MTC. However, exploring the role of calcitonin for this purpose was beyond the scope of the current study.

The ATA guidelines states that “contrast-enhanced CT of the neck and chest, three-phase contrast-enhanced multi-detector liver CT, or contrast-enhanced MRI of the liver, and axial MRI and bone scintigraphy are recommended in patients with extensive neck disease and signs or symptoms of regional or distant metastases, and in all patients with a serum calcitonin level greater than 500 pg/mL.” [9] Thus, preoperative imaging is warranted regardless of serum calcitonin levels when extensive neck disease or clinical signs of distant metastases are present. However, it does not define “extensive neck disease” or “signs and symptoms of regional metastases,” leaving room for interpretation. Furthermore, the most common presentation of sporadic MTC is a palpable neck lump without additional symptoms, observed in approximately 74% of cases [18]. Particularly for this large group of patients, determining the necessity of further imaging remains challenging. Although imaging modalities, particularly in the field of nuclear imaging, have advanced, there remains insufficient evidence to recommend PET/CT with various radiopharmaceuticals for staging MTC [19]. Consequently, multiple imaging modalities are required to accurately assess M1 disease at diagnosis, potentially leading to negative consequences for the patient.

Furthermore, when adhering to the 500 pg/mL calcitonin cutoff, relying solely on calcitonin levels can present several pitfalls. Calcitonin measurement is influenced by a range of pre-analytic and analytic factors, including interference from physiological and pathological conditions, instability at room temperature, and poor inter-method comparability [20-23]. Additionally, analytical challenges such as interference from heterophilic antibodies and macro-calcitonin can further compromise its reliability [24]. There are multiple studies investigating the role of preoperative calcitonin levels and different outcomes, such as recurrence and response to treatment [25-27]. However, literature on the association between preoperative calcitonin and M1 disease at diagnosis is relatively scarce. A study by Cohen et al reported in 2000 that a calcitonin level above 1000 pg/mL was significantly associated with M1 disease at diagnosis in 10.5% of cases vs 3.3% for a calcitonin level below 1000 pg/mL [28]. A study by Machens et al (2005) reported that M1 disease at diagnosis began appearing at preoperative calcitonin levels of 400 pg/mL and only in patients with pathologically proven lymph node metastases [25]. The current guideline recommendation is based on another study by Machens et al (2010), which examined the association between preoperative calcitonin levels and disease extent, primarily focusing on the extent of lymph node metastases in the neck [11]. In their cohort of 300 patients, 31 (12%) presented with M1 disease at diagnosis, and none had preoperative calcitonin levels below 500 pg/mL. A follow-up study by Park et al (2020) investigated the same question and additionally reported positive and negative LRs for different calcitonin cutoffs. In their cohort of 170 patients, 8 (4.7%) had M1 disease at diagnosis, with all having calcitonin levels above 500 pg/mL, except for one patient with a calcitonin level of 109 pg/mL [12]. In comparison, our cohort consisted of 2 patients with M1 disease who had calcitonin levels below 500 pg/mL. In the study by Park et al, all patients with M1 had pathologically confirmed N1b disease; however, no information was provided on the presence of suspicious lymph nodes on preoperative imaging. The reported positive LRs were generally low for most calcitonin cutoffs. Only at calcitonin cutoffs above 3000 pg/mL did the positive LR exceed 5, although it remained below 10, depicting moderate diagnostic value. Negative LRs were consistently above 0.2, indicating minimal to moderate diagnostic value. The positive and negative LRs reported in our study for different calcitonin cutoffs were in line with those in the study by Park et al. However, the negative LR for a 300 pg/mL calcitonin cutoff was lower, namely 0.0, indicating a strong diagnostic value.

As no single calcitonin cutoff appears optimal, this study explored multiple preoperative characteristics, to enhance the prediction of M1 disease at diagnosis. Using backward selection, we aimed to develop a multivariable model with both strong discriminatory ability as well as good clinical applicability. This resulted in a model combining preoperative calcitonin and suspicious lymph nodes on US. A multivariable model including calcitonin, lymph node status, and categorized tumor size resulted in an even higher AUC. Given the limited number of subjects and events, this study is likely underpowered to support a 3-variable model. Consequently, the robustness and generalizability of this finding should be assessed and validated in larger, future studies. Interestingly, pathologically proven lymph node metastases were a weaker predictor than suspicious lymph node metastases, likely reflecting the impact of FNA sampling errors.

Our study has several limitations. First, there is interobserver variability in US assessments, and visualization of central lymph nodes remains particularly challenging [29]. Compared to tertiary care centers, ultrasonographic detection of cervical lymph nodes in general clinical practice is considered less reliable [30]. However, since MTC is recommended to be managed in tertiary care centers with dedicated thyroid radiologists, this limitation is unlikely to be a significant issue. Although we were unable to investigate the differences between central and lateral lymph nodes in our analysis, it could still be valuable to distinguish central and lateral lymph nodes, as they may have different correlations with M1 disease. Second, the retrospective study design and single-center setting introduce potential selection and information biases that could affect the generalizability of our results. A possible cause of selection bias is the fact that we had to exclude patients without preoperative calcitonin measurement. The majority of patients with missing preoperative calcitonin values were those who underwent hemithyroidectomy after inconclusive FNA results, with MTC diagnosed postoperatively. Although unlikely, if these patients had high calcitonin levels but no M1 disease, or vice-versa, it could result in an overestimation of the association between calcitonin and M1 disease. Moreover, the association between suspicious lymph nodes and M1 disease at diagnosis could potentially be underestimated and thus be even stronger than the association we present in this study. This group is unlikely to have had suspicious lymph nodes on US, as any such findings would have led to further investigation, and a hemithyroidectomy would not have been performed. With regard to the prevalence of M1 disease, we reviewed this group and found that including them would only slightly reduce the prevalence of M1 disease to approximately 23%.

Due to the retrospective nature of our study, there is a risk of incomplete or inaccurate data potentially leading to information bias. However, we aimed to minimize this by utilizing a clinician-collected database with comprehensive information from electronic patient records, and not excluding patients with missing data. Instead, we employed multiple imputation to enable whole-case analysis. Imputation helps mitigate bias—particularly the bias introduced by complete case analysis—while preserving statistical power by maintaining the sample size.

Another limitation of our study is the long inclusion period, which encompasses significant advances in imaging modalities and evolving guidelines. This introduces the potential for inconsistencies when comparing data across different time periods. Nonetheless, the results remained consistent following sensitivity analyses that omitted all cases before 2006. Finally, there is a possibility that occult or micrometastases may have been missed at initial presentation. However, since most patients in our cohort had long-term follow-up, this could be verified. The presence of micrometastases in patients with NED during follow-up (44% of our cohort) is very unlikely. Additionally, all patients with biochemical incomplete response underwent long-term follow-up to check for the presence of M1 disease.

It is reasonable to question whether imaging to detect M1 disease should be performed routinely before, rather than after, thyroidectomy. If M1 detection does not substantially alter primary treatment, such imaging could be reserved for patients with persistently elevated postoperative calcitonin levels. However, we believe that preoperative knowledge of M1 disease may influence surgical strategy: in such patients, a more conservative approach may be appropriate, as extensive procedures like additional neck dissections for asymptomatic lymph nodes carry higher morbidity without clear benefit in the context of systemic disease. This approach aligns with the ATA guideline recommendations, which states that “in the presence of extensive regional or metastatic disease, less aggressive surgery in the central and lateral neck may be appropriate to preserve speech, swallowing, parathyroid function, and shoulder mobility,” and reflects the standard practice in our center [9]. Further research is needed to identify which patients benefit most from early M1 detection and how to incorporate this information into surgical planning to optimize an individually tailored approach.

Also, considerations of cost-effectiveness and variations in healthcare systems and clinical practice across countries are important when deciding to perform additional imaging or not, as such differences may limit the universal applicability of any imaging recommendation. However, these aspects were beyond the scope of the present study.

In any decision-making algorithm, the key question ultimately revolves around determining the acceptable probability of missing M1 disease. However, as the probability of M1 disease at diagnosis based on the current guideline recommendations has not been studied, there is inherent uncertainty in our current clinical practice. In this study, we demonstrate that the risk of having M1 disease at a calcitonin level of 500 pg/mL is already 14.1% if only calcitonin is used for prediction. Combining calcitonin levels with the presence of suspicious lymph nodes clearly differentiates the risk of M1 disease between patients with and without suspicious lymph nodes. Patients with suspicious lymph nodes exhibit significantly higher risks of M1 disease across all calcitonin levels, whereas patients without suspicious lymph nodes show an overall reduced risk. Should the field accept a 2.5% predicted probability of M1 disease as a clinically relevant threshold, a novel decision tree combining suspicious lymph nodes on US and preoperative calcitonin levels would suggest additional imaging for all patients with suspicious lymph nodes, as well as for those without suspicious lymph nodes but with calcitonin levels exceeding 180 pg/mL. This decision tree demonstrated substantially enhanced specificity compared to a model that utilizes calcitonin alone (29% vs 3%). However, as these results are based on a single study, further validation through a large multicenter study, which would allow investigation of multiple preoperative variables, is needed before drawing definitive conclusions regarding new cutoff values and a potential new decision tool.

In conclusion, our results may fuel future discussions on improving guideline recommendations. Specifically, performing additional imaging may be improved by incorporating the presence of suspicious lymph nodes on US into the decision-making algorithm, as this is the strongest predictor for M1 disease should our findings be validated in other cohorts.

## Data Availability

The data that support the findings of this study are available from the corresponding author on reasonable request.

## Abbreviations

ATA: American Thyroid Association
AUC: area under the curve
CEA: carcinoembryonic antigen
CT: computed tomography
FDG: fluorodeoxyglucose
FNA: fine needle aspiration
IQR: interquartile range
LR: likelihood ratio
M1: distant metastasis
MEN: multiple endocrine neoplasia
MRI: magnetic resonance imaging
MTC: medullary thyroid cancer
NED: no evidence of disease
NPV: negative predictive value
OR: odds ratio
PET: positron emission tomography
PPV: positive predictive value
ROC: receiver operating characteristic
US: ultrasound
